# Parents of children and adolescents knowledge, attitude, and practice toward metabolically healthy obesity: a cross-sectional survey

**DOI:** 10.3389/fpubh.2025.1591300

**Published:** 2025-10-24

**Authors:** Fengyu Zhong, Jingbo Feng, Lin Zhang, Lin Lu, Hui Yuan, Jing Li, Tian Tian, Youfang Hu

**Affiliations:** ^1^Department of Children’s Health Care, Jiangsu Province Hospital, Jiangsu Women and Children’s Health Hospital, Nanjing, Jiangsu, China; ^2^The First Clinical Medical College of Nanjing Medical University, Nanjing, Jiangsu, China; ^3^Nanjing Pukou People’s Hospital, Liangjiang Hospital Southeast University, Nanjing, Jiangsu, China

**Keywords:** children, adolescents, obesity, metabolically, obesity, health knowledge, attitude, practice, cross-sectional studies

## Abstract

**Introduction:**

Understanding parental knowledge, attitudes, and practices (KAP) regarding metabolically healthy obesity (MHO) in children and adolescents is crucial. This study aimed to assess the KAP levels of parents concerning MHO and identify factors influencing these levels.

**Methods:**

A cross-sectional study was conducted at Jiangsu Provincial People’s Hospital (June 28, 2023–August 3, 2023). 534 valid questionnaires were collected. Data analysis included descriptive statistics, univariable/multivariable regression, and Spearman correlation to determine KAP levels and influencing factors. Cronbach’s *α* was 0.890.

**Results:**

Median scores were 11 (IQR: 7–13; maximum 18) for knowledge, 32 (IQR: 30–35; maximum 40) for attitude, and 32 (IQR: 27–35; maximum 40) for practice. Significant positive correlations were found between knowledge and attitude (*r* = 0.407, *p* < 0.001), knowledge and practice (*r* = 0.357, *p* < 0.001), and attitude and practice (*r* = 0.456, *p* < 0.001). Non-medical occupations (OR = 0.658, *p* = 0.037) and income 8,000–12,000 (OR = 2.796, *p* = 0.001) were linked to knowledge. Income 5,000–8,000 (OR = 2.864, *p* = 0.001) and 8,000–12,000 (OR = 2.392, *p* = 0.013) related to attitude, while income 8,000–12,000 (OR = 2.798, *p* = 0.001) and >12,000 (OR = 2.340, *p* = 0.013) related to practice.

**Conclusion:**

Parents showed moderate knowledge but good attitudes/practices toward MHO. KAP dimensions were positively correlated. Socioeconomic status, particularly income, significantly associated with knowledge/practice.

## Introduction

Overweight and obesity are emerging as a global public health crisis and are related to the rapid economic growth and changes in lifestyles (1, 2). Obesity and overweight have more than doubled in the past 30 years, with obesity alone affecting over 1 billion people globally as of 2024 (3). China has the largest number of overweight and obese individuals worldwide, and approximately 20% of Chinese children and adolescents have excess weight (4). Obesity not only impacts the normal growth and development of children and adolescents but also stands as a primary risk factor for non-communicable diseases in this age group, including cardiovascular diseases, type 2 diabetes mellitus (T2DM), musculoskeletal disorders, cancer, and mental health issues, leading to an increased likelihood of premature death and disability in adulthood (5, 6).

Still, not all overweight/obese individuals are equal, and research has revealed that individuals with obesity who have unhealthy metabolic profiles are more prone to cardiovascular diseases and overall mortality than those with a healthier metabolic profile (7, 8). Hence, it has been recommended to classify obesity into distinct phenotypes based on body mass index (BMI) and metabolic status, including metabolically healthy obesity (MHO) and metabolically unhealthy obesity (MUO), and to adopt different management strategies (9, 10). People with MHO do not appear to have cardiometabolic abnormalities despite a BMI in the obesity range, while patients with MUO display T2DM, fatty liver disease, hypertension, dyslipidemia, and cardiovascular diseases (10). In 2019, China issued an expert consensus that clearly defines MHO as individuals whose BMI qualifies as obese but does not exhibit metabolic abnormalities such as high blood pressure, high blood glucose, lipid disorders, or insulin resistance (11). Still, the data regarding the overall prevalence of MHO and MUO in Chinese children and adolescents according to the criteria mentioned above is poorly known.

“The ‘Healthy China 2030’ Blueprint,” the “Healthy Children Action Enhancement Plan (2021–2025),” and the “China Children’s Development Program (2021–2030)” all emphasize the need to address and control childhood obesity from a national strategic perspective (12). Identifying the risk factors for childhood and adolescent obesity is a crucial approach to preventing and managing obesity, and the concept of MHO brings new insights to the treatment and management of childhood obesity (13). Currently, the principles of treating childhood and adolescent obesity involve reducing energy intake and increasing energy expenditure to reduce body fat while promoting normal growth and health in the affected children (14–16). Treatment methods primarily encompass (1) lifestyle interventions, (2) psychological and behavioral interventions, (3) medication, and (4) metabolic weight reduction surgery. Lifestyle interventions, which include diet, exercise, and sleep management, should be family-based, integrated into daily life, and involve the active participation of children, parents, teachers, and healthcare professionals, with a duration of at least 1 year (14–16). Previous studies commonly acknowledge that genetic and environmental factors contribute to obesity (17–19). Therefore, the World Health Organization (WHO) specifically urges improvements in children’s and adolescents’ obesity through parental behaviors, early life conditions, individual diet, and physical activity (20, 21). Parents play a crucial role in recognizing and managing childhood and adolescent obesity, through early recognition and health monitoring, modeling and shaping healthy behaviors, engagement in behavior change interventions, and long-term maintenance (22, 23). Even among children with MHO, parents should ensure the maintenance of protective behaviors (high physical activity, healthy diet, reduced sedentary time). Regular health check-ups are needed to detect any changes in metabolic profile. Encouraging healthy habits, such as high fruit/vegetable intake and low screen time, directly relates to a more favorable metabolic profile and slower progression to metabolic syndrome (24).

There is currently limited direct research specifically focused on parental awareness, attitudes, and behaviors regarding metabolically healthy obesity (MHO) in children and adolescents. Most existing studies address parental perceptions, knowledge, and practices concerning childhood obesity more broadly, with only indirect relevance to the MHO phenotype (25–27). Therefore, this study aims to explore the levels of parental awareness, attitudes, and behaviors regarding MHO in children and adolescents, which holds significant implications for preventing and managing obesity-related health issues. Particularly in the context of the escalating obesity epidemic, understanding parental perceptions and actions toward MHO provides valuable insights for devising effective health education and intervention strategies. Moreover, the study can unveil factors influencing parental awareness and behavioral practices, including socioeconomic status, thereby offering more targeted recommendations for future health promotion initiatives. This research holds crucial practical and policy implications for fostering the health development of children and adolescents, as well as preventing and managing obesity-related health problems.

## Materials and methods

The study was reported according to the Strengthening the Reporting of Observational Studies in Epidemiology (STROBE) checklist (28).

### Study design and participants

This cross-sectional study was conducted at Jiangsu Provincial People’s Hospital from June 28, 2023, to August 3, 2023. Established in 1936 as the affiliated clinic to Jiangsu Provincial Medical College, Jiangsu Provincial People’s Hospital is the largest comprehensive hospital in Jiangsu Province. It is designated as a Grade 3A hospital, representing the highest tier in China’s hospital classification system for comprehensive service and advanced medical capabilities. It houses approximately 4,200 beds with a daily patient volume approaching 9,000. The staff exceeds 6,800 employees, including specialists in multiple nationally and provincially recognized key clinical disciplines. The hospital serves the population of Nanjing municipality and the wider Jiangsu Province, regions that include both highly urbanized areas and vast rural communities. Jiangsu itself is one of China’s most economically developed provinces, but it exhibits strong urban–rural socioeconomic diversity and health inequality. Over 95% of the population has basic medical security, but out-of-pocket health spending remains a significant burden for the lowest-SES and high-cost families, especially those with chronic diseases and older adults.

The participants were the parents of children and adolescents, enrolled at the General outpatient clinic of child health department (i.e., obese/overweight patients found in general physical examination screening), the pediatric nutrition clinic, and the pediatric child weight management clinic (i.e., patients who are seen for obesity). The study was approved by the Medical Ethics Committee of Jiangsu Provincial People’s Hospital. Each participant provided written informed consent before completing the questionnaire.

The inclusion criteria were (1) parents of children or adolescents who could be encountered in routine outpatient work, (2) individuals with clear consciousness, normal cognitive function, and the ability to complete the questionnaire (in Chinese) without communication barriers, and (3) participants who provided informed consent for this study and voluntarily agreed to participate. The exclusion criteria were (1) participants who did not consent to participate in the study, (2) respondents with completion time >1,800 s or < 150 s (the minimum time to answer the questionnaire was calculated as 2 s for a single-choice question and 3 s for a multiple-choice question; the maximum was set arbitrarily to exclude those who might be checking for answers on the internet or other sources), (3) incomplete or duplicate questionnaire responses, or (4) questionnaires with logical errors in the responses.

### Questionnaires and data collection

Since no questionnaire was available to answer the research question in the specific study population, a new KAP questionnaire was designed based on the literature (10, 29, 30) and the Expert Consensus on the Definition and Screening of Metabolically Healthy Obesity in Chinese Children (11). The questionnaire was refined based on the feedback of 15 experts in pediatric endocrinology, child growth and development, and the child healthcare field. A pilot study was conducted with 49 participants, and the Cronbach’s *α* coefficient was 0.872.

The questionnaire consisted of four sections (Supplementary materials): demographic characteristics (11 items), knowledge (15 items), attitudes (eight items), and practices (eight items) related to MHO. The scores were computed individually for each subcategory. In the knowledge section, the responses were coded as 1 = correct, 0 = incorrect, and 0 = uncertain. The attitude section used a five-point Likert scale, ranging from “totally agree” (5 points) to “totally disagree” (1 point). The scores for negative topics were reverse-coded before calculation. The practice section also used a five-point Likert scale, ranging from “always” (5 points) to “never” (1 point). Hence, the total scores for the knowledge, attitude, and practice dimensions were 0–15, 8–40, and 8–40, respectively.

Data was collected through an online questionnaire using a QR code generated with the WeChat-based Questionnaire Star applet. The parents were approached for participation when they visited the hospital. Data are collected using the Questionnaire Star applet. The participants accessed the questionnaire by scanning the QR code via WeChat and completing it. The questionnaire tool 2–30 min to complete. The research assistants were available to provide guidance and answer questions when the questionnaire was completed on-site. Once enrolled and the QR code was scanned, the participants could complete the questionnaire at their leisure. All items were mandatory. A given IP address could be used only once to submit a questionnaire.

### Sample size

The sample size calculation was performed using the Open Epi calculator tool, using the following formula (31, 32):


$$n=\frac{p\times (1-p)\times z^{2}}{e^{2}}$$

where *n* is the required sample size, *z* is the *Z* statistic for the confidence level (e.g., 1.96 for a 95% confidence level or 2.58 for a 99% confidence level), *p* is the estimated proportion (generally set at 0.5), and e is the margin of error (typically set at 5%). A smaller margin of error increases the reliability of the conclusions. Hence, using the formula.


$$n=\frac{0.5\times (1-0.5)\times 1.96^{2}}{0.05^{2}}=384.16$$

a minimum of 384 valid questionnaires were needed to be collected to ensure the required level of confidence in the research findings.

### Statistical analysis

The data were analyzed using SPSS 22.0 (IBM, Armonk, NY, USA). The continuous data were tested for normal distribution using the Kolmogorov–Smirnov test. The continuous data following the normal distribution were presented as means ± standard deviations and analyzed using Student’s *t*-test or ANOVA. Those with a non-normal distribution were presented as medians [interquartile ranges (IQRs)] and analyzed using the Mann–Whitney *U*-test or Wilcoxon’s test. Categorical data were presented as *n* (%) and analyzed using the chi-squared test. Correlations between scores were analyzed using Pearson correlation coefficients (normal distribution) or Spearman correlation coefficients (non-normal distribution). Univariate logistic regression analyses were performed to determine the factors associated with the KAP scores. Variables with *p*-values < 0.10 were included in the multivariable logistic regression analyses. In this study, the *p*-values were reported with three decimals, and *p*-values < 0.05 were considered statistically significant.

## Results

### Demographics characteristics

This study collected 632 questionnaires, but three participants did not agree to the use of the collected data for scientific research, and 95 questionnaires took less than 150 s or more than 1,800 s to fill in. Therefore, 534 questionnaires were included in the analysis. The results confirmed the questionnaire’s good reliability and validity, with a Cronbach’s *α* coefficient of 0.890 and a Kaiser-Meyer-Olkin (KMO) value of 0.893. The Cronbach’s α coefficients for knowledge, attitudes, and practices were 0.868, 0.723, and 0.882, respectively. The knowledge, attitude, and practice scores did not meet the normal distribution (Supplementary Table S1).

The characteristics of the participants are shown in Table 1. Most participants were female (75.5%), aged 31–39 years (68.0%), had a college/undergraduate education (72.5%), were living in urban areas (94.4%), were working in non-medical occupations (65.2%), had a monthly income of 5,000–8,000 RMB (33.5%), were married (96.4%), had one child or adolescent in the household (68.7%), and showed no family history of obesity (79.8%). The primary caregiver of the child or adolescent was the mother (65.5%).

**Table 1:** Characteristics of the participants.

Variables	*n* (%)	Knowledge		Attitude		Practice	
		Median (25th, 75th percentile)	*p*	Median (25th, 75th percentile)	*p*	Median (25th, 75th percentile)	*p*
Total	534	11 (7, 13)		32 (30, 35)		32 (27, 35)	
Gender			0.076		0.271		0.548
Male	131 (24.5)	11 (7, 14)		32 (29, 34)		31 (26, 35)	
Female	403 (75.5)	11 (6, 13)		32 (30, 35)		32 (27, 36)	
Age (years)			0.282		0.188		0.663
30 and below	44 (8.2)	10 (7, 12)		31.5 (27, 34)		32 (24.5, 35.5)	
31–39	363 (68.0)	11 (6, 14)		32 (30, 35)		32 (27, 36)	
41–49	104 (19.5)	11 (8, 14)		32 (30, 35.5)		32 (26, 34.5)	
50 and above	23 (4.3)	10 (7, 13)		31 (28, 35)		31 (24, 34)	
Education level			< 0.001		< 0.001		< 0.001
Junior high school or below	18 (3.4)	3 (0, 10)		31 (28, 34)		28 (24, 34)	
High school/technical school	51 (9.6)	8 (1, 11)		30 (27, 33)		27 (23, 32)	
College/undergraduate	387 (72.5)	11 (7, 13)		32 (29, 34)		32 (27, 35)	
Graduate and above	78 (14.6)	13 (9, 15)		34 (31, 37)		33 (29, 39)	
Residence			0.001		0.032		0.013
Urban	504 (94.4)	11 (7, 14)		32 (30, 35)		32 (27, 35)	
Rural	30 (5.6)	8 (4, 10)		30.5 (28, 34)		28.5 (24, 32)	
Occupation			< 0.001		< 0.001		0.001
Medical field-related	178 (33.3)	12 (9, 14)		33 (31, 37)		32 (28, 37)	
Other occupations	348 (65.2)	10 (5, 13)		32 (29, 34)		31 (26, 35)	
Retired	8 (1.5)	9 (4.5, 11.5)		28.5 (26.5, 33.5)		25.5 (22.5, 30.5)	
Monthly income (RMB)			< 0.001		< 0.001		< 0.001
< 5,000	80 (15.0)	7 (2.5, 11)		30.5 (27, 33)		27.5 (23.5, 33)	
5,000–8,000	179 (33.5)	10 (7, 13)		32 (29, 34)		30 (26, 34)	
8,000–12,000	164 (30.7)	12 (9, 14)		33 (30, 36)		32 (28.5, 37)	
> 12,000	111 (20.8)	12 (8, 14)		33 (30, 36)		33 (28, 36)	
Marital status			0.306		0.517		0.492
Unmarried	14 (2.6)	8.5 (2, 13)		31 (27, 34)		32 (25, 36)	
Married	515 (96.4)	11 (7, 14)		32 (30, 35)		31 (27, 35)	
Divorced/widowed	5 (0.9)	9 (7, 11)		30 (29, 32)		37 (32, 40)	
Number of children and adolescents in the household			0.042		0.276		0.904
1	367 (68.7)	11 (7, 14)		32 (29, 34)		32 (27, 35)	
2	158 (29.6)	10 (5, 13)		32 (30, 36)		31 (26, 36)	
3 or more	9 (1.7)	6 (0, 13)		32 (27, 34)		32 (21, 37)	
Primary caregiver for the children and adolescents			0.821		0.405		0.638
Father	126 (23.6)	10 (6, 14)		31.5 (29, 34)		31 (27, 35)	
Mother	350 (65.5)	11 (7, 13)		32 (30, 35)		32 (27, 35)	
Grandfather/grandmother	40 (7.5)	10 (6, 14)		33 (29.5, 34)		32 (24, 35.5)	
Maternal/paternal grandparents	17 (3.2)	7 (2, 15)		32 (29, 34)		32 (27, 36)	
Nanny	1 (0.2)	9 (9, 9)		36 (36, 36)		40 (40, 40)	
Relationship with household children			0.068		0.053		0.382
Father	128 (24.0)	11.5 (7.5, 14)		32 (29, 34)		31.5 (27, 35)	
Mother	393 (73.6)	11 (6, 13)		32 (30, 35)		32 (27, 36)	
Grandfather/grandmother	11 (2.1)	7 (3, 12)		29 (26, 33)		29 (22, 32)	
Maternal/paternal grandparents	2 (0.4)	11.5 (9, 14)		34 (31, 37)		34.5 (34, 35)	
Family history of obesity			0.053		0.467		0.835
Yes	108 (20.2)	11 (8, 14)		32 (30, 35)		32 (27, 35)	
No	426 (79.8)	11 (6, 13)		32 (29, 35)		31 (26, 35)	

### Knowledge

The median knowledge score was 11 (61.1%; IQR: 7–13; range: 0–18) (Supplementary Table S2). Higher knowledge scores were observed with higher education (*p* < 0.001), urban residence (*p* = 0.001), medical occupation (*p* < 0.001), higher income (*p* < 0.001), and only one child or adolescent in the household (*p* = 0.042) (Table 1). The item with the highest score was K1 (88.0%; “Overweight and obesity are defined as the excessive or abnormal accumulation of fat that poses health risks, and they are chronic metabolic diseases caused by the interaction of various factors, including genetics and the environment.”), while the item with the lowest score was K7 (13.9%; “MHO, as a form of obesity with normal metabolism, is no different from other health states”) (Supplementary Table S3).

### Attitudes

The median attitude score was 32 (80.0%; IQR: 30–35; range: 16–40) (Supplementary Table S2). Higher attitude scores were observed with higher education level (*p* < 0.001), urban residence (*p* = 0.032), medical occupation (*p* < 0.001), and higher income (*p* < 0.001) (Table 1). The item with the highest score was A3 (87.0%; “I believe it is necessary to control and improve the diet of children and adolescents to manage MHO.”), while the lowest score was seen for A6 (46.7%; “I believe MHO will resolve naturally or transform into metabolically healthy normal-weight individuals as children and adolescents develop.”) (Supplementary Table S4).

### Practices

The median practice score was 32 (80.0%; IQR: 27–35; range: 8–40) (Supplementary Table S2). Higher practice scores were seen with higher education level (*p* < 0.001), urban residents (*p* = 0.013), medical occupation (*p* = 0.001), and a higher income (*p* < 0.001) (Table 1). The highest practice score was observed with P5 (79.2%; “I will educate MHO children and adolescents to develop healthy sleep hygiene habits, such as maintaining a regular sleep schedule and avoiding stimulating activities before bedtime.”) and the lowest score was seen with P8 (53.3%; “I will collaborate with teachers and healthcare professionals to participate in intervention programs for MHO children and adolescents.”) (Supplementary Table S5).

### Correlations

The knowledge scores were positively correlated with the attitude (*r* = 0.407, *p* < 0.001) and practice (*r* = 0.357, *p* < 0.001) scores, while the attitude scores were positively correlated to the practice scores (*r* = 0.456, *p* < 0.001) (Table 2).

**Table 2:** Correlation analysis.

Dimensions	Knowledge	Attitude	Practice
Knowledge	1.000		
Attitude	0.407 (*p* < 0.001)	1.000	
Practice	0.357 (*p* < 0.001)	0.456 (*p* < 0.001)	1.000

### Multivariable analyses

All multivariable analyses used the median score as the cutoff value. Non-medical occupations (OR = 0.658, 95%CI: 0.442–0.976, *p* = 0.037) and income 8,000–12,000 (OR = 2.796, 95%CI: 1.487–6.355, *p* = 0.001) were independently associated with the knowledge scores (Table 3). Income 5,000–8,000 (OR = 2.864, 95%CI: 1.388–24.830, *p* = 0.001) and income 8,000–12,000 (OR = 2.392, 95%CI: 1.421–11.962, *p* = 0.013) were independently associated with the attitude scores (Table 4). Income 8,000–12,000 (OR = 2.798, 95%CI: 1.498–5.336, *p* = 0.001) and income >12,000 (OR = 2.340, 95%CI: 1.195–4.666, *p* = 0.013) were independently associated with the practice scores (Table 5).

**Table 3:** Univariable and multivariable regression analysis of knowledge.

Cut-off value: ≥11/<11	No.	Univariable		Multivariable (enter method)	
		OR (95%CI)	*p*	OR (95%CI)	*p*
Gender					
Male	73/131	ref.			
Female	202/403	0.798 (0.537, 1.187)	0.266		
Age (years) (comparison method = difference)					
30 and below	19/44	ref.			
31–39	187/363	1.398 (0.744, 2.628)	0.298		
41–49	59/104	1.459 (0.885, 2.406)	0.139		
50 and above	10/23	0.755 (0.319, 1.785)	0.522		
Education level (comparison method = difference)					
Junior high school or below	4/18	ref.		ref.	
High school/technical school	15/51	1.458 (0.412, 5.162)	0.599	1.067 (0.318, 4.072)	0.919
College/undergraduate	206/387	3.299 (1.700, 6.400)	< 0.001	1.538 (0.510, 5.423)	0.456
Graduate and above	50/78	3.477 (1.853, 6.523)	< 0.001	1.933 (0.566, 7.520)	0.300
Residence					
Urban	269/504	ref.		ref.	
Rural	6/30	0.218 (0.088, 0.543)	0.001	0.413 (0.151, 1.005)	0.051
Occupation					
Medical field-related	111/178	ref.		ref.	
Other occupations	164/356	0.506 (0.353, 0.730)	< 0.001	0.658 (0.442, 0.976)	0.037
Monthly income (RMB) (comparison method = difference)					
< 5,000	22/80	ref.		ref.	
5,000–8,000	86/179	2.438 (1.376, 4.318)	0.002	1.808 (0.999, 3.334)	0.051
8,000–12,000	104/164	2.927 (1.909, 4.487)	< 0.001	2.796 (1.487, 5.355)	0.001
> 12,000	63/111	1.549 (1.004, 2.392)	0.048	1.958 (0.990, 3.929)	0.054
Marital status					
Unmarried	7/19	0.584 (0.213, 1.515)	0.278		
Married	268/515	ref.			
Number of children and adolescents in the household (comparison method = difference)					
1	197/367	ref.			
2	75/158	0.780 (0.537, 1.133)	0.192		
3 or more	3/9	0.489 (0.121, 1.978)	0.316		
Primary caregiver for the children and adolescents					
Father	62/126	ref.			
Mother	187/350	1.184 (0.788, 1.780)	0.416		
Grandfather/grandmother	19/40	0.934 (0.458, 1.904)	0.851		
Maternal/paternal grandparents	7/17	0.723 (0.259, 2.018)	0.535		
Relationship with household children					
Father	73/128	ref.		ref.	
Mother	198/393	0.765 (0.512, 1.144)	0.192	0.826 (0.540, 1.260)	0.376
Grandfather/grandmother	4/13	0.333 (0.092, 1.094)	0.081	0.610 (0.161, 2.070)	0.433
Family history of obesity					
Yes	60/108	ref.			
No	215/426	0.815 (0.533, 1.246)	0.345		

**Table 4:** Univariable and multivariable regression analysis of attitude.

Cut-off value: ≥32 /<32	No.	Univariable		Multivariable (enter method)	
		OR (95%CI)	*p*	OR (95%CI)	*p*
Gender					
Male	68/131	ref.			
Female	232/403	1.257 (0.846, 1.867)	0.257		
Age (years) (comparison method = difference)					
30 and below	22/44	ref.			
31–39	210/363	1.373 (0.734, 2.568)	0.322		
41–49	58/104	1.076 (0.654, 1.771)	0.772		
50 and above	10/23	0.641 (0.271, 1.514)	0.310		
Education level (comparison method = difference)					
Junior high school or below	8/18	ref.		ref.	
High school/technical school	17/51	0.625 (0.209, 1.872)	0.401	0.820 (1.880, 0.730)	0.753
College/undergraduate	217/387	2.018 (1.125, 3.619)	0.018	1.365 (1.783, 1.713)	0.591
Graduate and above	58/78	3.628 (1.933, 6.810)	< 0.001	1.934 (1.899, 2.798)	0.304
Residence					
Urban	289/504	ref.		ref.	
Rural	11/30	0.431 (0.201, 0.924)	0.031	0.628 (1.575, 0.359)	0.306
Occupation					
Medical field-related	119/178	ref.		ref.	
Other occupations	181/356	0.51 9 (0.356, 0.756)	0.001	0.752 (1.222, 0.241)	0.155
Monthly income (RMB) (comparison method = difference)					
< 5,000	33/80	ref.		ref.	
5,000–8,000	93/179	1.540 (0.904, 2.625)	0.112	1.765 (1.363, 6.280)	0.066
8,000–12,000	103/164	1.938 (1.281, 2.931)	0.002	2.864 (1.388, 24.830)	0.001
> 12,000	71/111	1.634 (1.053, 2.535)	0.028	2.392 (1.421, 11.962)	0.013
Marital status					
Unmarried	9/19	0.617 (0.231, 1.587)	0.315		
Married	291/515	ref.			
Number of children and adolescents in the household (comparison method = difference)					
1	205/367	ref.			
2	90/158	1.046 (0.718, 1.524)	0.815		
3 or more	5/9	0.966 (0.256, 3.645)	0.959		
Primary caregiver for the children and adolescents					
Father	63/126	ref.			
Mother	203/350	1.381 (0.918, 2.078)	0.122		
Grandfather/grandmother	23/40	1.353 (0.660, 2.773)	0.409		
Maternal/paternal grandparents	10/17	1.429 (0.512, 3.990)	0.496		
Relationship with household children					
Father	67/128	ref.			
Mother	229/393	1.271 (0.852, 1.898)	0.240		
Grandfather/grandmother	4/13	0.401 (0.107, 1.306)	0.249		
Family history of obesity					
Yes	62/108	ref.			
No	238/426	0.939 (0.613, 1.439)	0.773		

**Table 5:** Univariable and multivariable regression analysis of practice.

Cut-off value: ≥32 /<32	No.	Univariable		Multivariable (enter method)	
		OR (95%CI)	*p*	OR (95%CI)	*p*
Gender					
Male	65/131	ref.			
Female	204/403	1.041 (0.702, 1.544)	0.842		
Age (years) (comparison method = difference)					
30 and below	23/44	ref.			
31–39	182/363	0.918 (0.491, 1.717)	0.789		
41–49	53/104	0.990 (0.603, 1.626)	0.969		
50 and above	11/23	0.876 (0.373, 2.059)	0.762		
Education level (comparison method = difference)					
Junior high school or below	5/18	ref.		ref.	
High school/technical school	14/51	0.984 (0.296, 3.269)	0.979	0.799 (0.248, 2.783)	0.713
College/undergraduate	198/387	2.746 (1.459, 5.170)	0.002	1.303 (0.454, 4.158)	0.630
Graduate and above	52/78	3.744 (2.011, 6.970)	<0.001	1.829 (0.559, 6.525)	0.322
Residence					
Urban	261/504	ref.		ref.	
Rural	8/30	0.339 (0.148, 0.775)	0.010	0.652 (0.263, 1.508)	0.323
Occupation					
Medical field-related	107/178	ref.		ref.	
Other occupations	162/356	0.565 (0.391, 0.815)	0.002	0.755 (0.511, 1.114)	0.157
Monthly income (RMB) (comparison method = difference)					
< 5,000	22/80	ref.		ref.	
5,000–8,000	81/179	2.179 (1.230, 3.862)	0.008	1.734 (0.961, 3.187)	0.068
8,000–12,000	101/164	2.863 (1.871, 4.381)	<0.001	2.798 (1.498, 5.336)	0.001
> 12,000	65/111	1.777 (1.149, 2.748)	0.010	2.340 (1.195, 4.666)	0.013
Marital status					
Unmarried	12/19	1.587 (0.610, 4.348)	0.354		
Married	257/515	ref.			
Number of children and adolescents in the household (comparison method = difference)					
1	186/367	ref.			
2	77/158	0.925 (0.637, 1.343)	0.682		
3 or more	6/9	2.024 (0.500, 8.193)	0.323		
Primary caregiver for the children and adolescents					
Father					
Mother	58/126	ref.			
Grandfather/grandmother	176/350	1.186 (0.788, 1.784)	0.413		
Maternal/paternal grandparents	24/40	1.759 (0.853, 3.624)	0.126		
Relationship with household children					
Father	64/128	ref.			
Mother	199/393	1.026 (0.688, 1.529)	0.901		
Grandfather/grandmother	6/13	0.861 (0.259, 2.718)	0.792		
Family history of obesity					
Yes	60/108	ref.			
No	209/426	0.771 (0.504, 1.178)	0.229		

## Discussion

This cross-sectional study analyzed the KAP levels of parents of children and adolescents regarding MHO. The KAP dimensions were positively correlated to each other. Socioeconomic status was associated with knowledge and practice scores.

The present study showed moderate parental knowledge (61.1%) toward MHO but positive attitudes and proactive practice (both 80.0%). No previous studies examined the KAP of parents toward MHO specifically. Available studies mostly examined obesity in general and only brushed the subject of MHO (25–27). Previous studies among Chinese university students showed poor knowledge of obesity and its complications (33, 34). Straughan and Xu showed that the parental KAP toward childhood obesity was moderate in Singapore (35). Similar results were observed in Congo (36) and even in developed countries like the United States of America (37). In the present study, participants with non-medical occupations had lower knowledge of MHO. Indeed, medical workers are more likely to have learnt about MHO in the course of their occupation or continuing education. Healthcare providers are primary sources of medical and health information for patients and the general population. Studies showed that the KAP toward obesity can be low or moderate among healthcare providers in specific parts of the world (38). The KAP of healthcare providers was not examined in the present study, and future studies should involve such professionals. Indeed, improving the parental KAP could involve improving the KAP upstream, especially given that parent-physician communication plays an important role in KAP toward obesity and its prevention/management (39).

Socioeconomic conditions are well-known to be related to health literacy, health risk behaviors, and health status (40). Accordingly, in the present study, parents with a higher income had higher knowledge scores, as did those involved in medical professions. Only a higher income was associated with better attitude and practice scores. In the study area, an income of < 5,000 yuan is a lower income level, covering some graduates, grassroots service workers, or part-time workers who have just joined the workforce. An income of 5,000–8,000 yuan is a lower middle/mainstream wage, which is the typical income range of white-collar workers and ordinary employees in many cities, and is the backbone of society, but life pressure may be greater. An income of 8,000–12,000 yuan is a middle/upper middle income, usually for more experienced professionals, grassroots managers, or technical backbones with relatively affordable lives. An income of > 12,000 yuan represents the high-income group, including senior managers, senior experts, business owners, etc., with strong consumption and savings ability. Those results are globally supported by Straughan and Xu (35), who showed that parents with higher KAP scores toward obesity were younger, had a higher income, were not working full-time, and were living in a separate household from the children’s grandparents. Similar results were also reported in various countries (41–43). Nevertheless, all three dimensions were positively correlated to each other. Based on the KAP theory, knowledge is the basis for practice, but attitude is the force driving practice (44, 45). Hence, improving knowledge and attitudes through educational and motivational interventions could also improve practice (46, 47). Future studies should also examine such interventions.

This study had limitations. The study met the calculated sample size, but it was performed at a single center and thus represents only a small proportion of the parents in China, limiting generalizability. The study was cross-sectional and, therefore, represents a single point in time, preventing causality analysis and the changes over time. Nevertheless, the results could serve as a historical baseline to evaluate the effect of future educational activities on MHO. All KAP studies are at risk of social desirability bias, i.e., the participants might be tempted to answer what they know they should think/do instead of what they are actually thinking/doing (48, 49). Considering the high attitude and practice scores, that bias is a possibility. Future studies should involve multiple centers and cover larger areas of the country. Finally, the sparse effect raises the odds ratio’s confidence interval. It refers to the phenomenon that when the sample size is small, and there are many variables, the parameter estimation in the so-called “separated data” tends to be infinite (50–52). We acknowledge that the statistical analysis may have resulted in some inflation of the Type I error rate. However, given the exploratory nature of this study, the primary aim was hypothesis generation rather than definitive hypothesis testing. Accordingly, the acceptance of a slightly elevated risk of false-positive findings was considered justifiable, with the understanding that these results require confirmation in future, adequately powered confirmatory studies (53, 54). Therefore, pending confirmation, the results should be interpreted with caution.

## Conclusion

In conclusion, this study showed that the parents of children and adolescents had moderate knowledge but good attitudes and practices toward MHO. The KAP dimensions were positively correlated to each other. The socioeconomic status (living in an urban area, income, and working in a medical profession) was generally associated with the KAP scores. The results could be used to optimize educational strategies for parents and provide guidance and references for preventing and managing childhood and adolescent obesity. Policymakers should be made aware of the results and participate in the development of an intervention to improve the KAP of the parents toward MHO. Better practice could translate into patient outcomes.

## Data Availability

The original contributions presented in the study are included in the article/Supplementary material, further inquiries can be directed to the corresponding author.
