# Supplementary material for: Parents of children and adolescents knowledge, attitude, and practice toward metabolically healthy obesity: a cross-sectional survey
Source: Front Public Health. 2025 Oct 24;13:1591300. doi: 10.3389/fpubh.2025.1591300 (PMC12592141; doi:10.3389/fpubh.2025.1591300)
Supplement: Supplementary file 1 [file Supplementary_file_1.docx]

**SUPPLEMENTARY MATERIALS**

**Table S1.** Evaluation of the normal distribution for the KAP scores

|  | Kolmogorov-Smirnov normality test |
| --- | --- |
| Knowledge | P<0.05 |
| Attitude | P<0.05 |
| Practice | P<0.05 |

**Table S2.** KAP scores

|  | Median | 25th Percentile | 75th Percentile | Minimum | Maximum | <Median, n (%) | ≥Median, n (%) |
| --- | --- | --- | --- | --- | --- | --- | --- |
| Knowledge | 11 | 7 | 13 | 0 | 18 | 259 (48.5) | 275 (51.5) |
| Attitude | 32 | 30 | 35 | 16 | 40 | 234 (43.8) | 300 (56.2) |
| Practice | 32 | 27 | 35 | 8 | 40 | 265 (49.6) | 269 (50.4) |

**Table S3.** Knowledge items

| Item | a. Correct | b. Incorrect | c. Uncertain |
| --- | --- | --- | --- |
| 1. Overweight and obesity are defined as the excessive or abnormal accumulation of fat that poses health risks, and they are chronic metabolic diseases caused by the interaction of various factors, including genetics and the environment. (T) | 470 (88.0) | 9 (1.7) | 55 (10.3) |
| 1. Metabolically healthy obesity (MHO) refers to a condition in which a person’s body mass index (BMI) meets the criteria for obesity but is not accompanied by metabolic abnormalities. (T) | 381 (71.3) | 36 (6.7) | 117 (21.9) |
| 1. There are three screening indicators for MHO: blood pressure, fasting blood glucose, and triglycerides. (F) | 317 (59.4) | 20 (3.7) | 197 (36.9) |
| 1. Compared to adults, MHO is more common in children and adolescents. (T) | 279 (52.2) | 65 (12.2) | 190 (35.6) |
| 1. There is a gender difference in the prevalence of metabolic health and metabolic unhealthy obesity in Chinese children and adolescents, with girls having a higher incidence. (F) | 121 (22.7) | 138 (25.8) | 275 (51.5) |
| 1. Important risk factors for MHO in children and adolescents include being an only child, parental smoking, parental history of diseases (overweight, hypertension, diabetes), cesarean section delivery, premature birth, delayed delivery, high birth weight, insufficient sleep, and excessive screen time. (T) | 365 (68.4) | 31 (5.8) | 138 (25.8) |
| 1. MHO, as a form of obesity with normal metabolism, is no different from other health states. (F) | 74 (13.9) | 286 (53.6) | 174 (32.6) |
| 1. The MHO phenotype exhibits some instability and is prone to transitioning to metabolically unhealthy. (T) | 311 (58.2) | 31 (5.8) | 192 (36.0) |
| 1. Characteristics of MHO Population Compared to Metabolically Unhealthy Obesity (MUO) Population: (1) Lower liver and visceral fat content (T) | 198 (37.1) | 138 (25.8) | 198 (37.1) |
| (2) Lower subcutaneous fat content in the legs (F) | 142 (26.6) | 170 (31.8) | 222 (41.6) |
| (3) Stronger cardiorespiratory fitness and physical activity (T) | 169 (31.6) | 165 (30.9) | 200 (37.5) |
| (4) Lower insulin sensitivity (F) | 179 (33.5) | 114 (21.3) | 241 (45.1) |
| (5) Lower levels of inflammatory markers (T) | 172 (32.2) | 106 (19.9) | 256 (47.9) |
| (6) Normal functioning of adipose tissue (T) | 189 (35.4) | 151 (28.3) | 194 (36.3) |
| 1. The MHO population has the same cardiovascular disease incidence as the metabolically healthy normal-weight population. (F) | 122 (22.8) | 258 (48.3) | 154 (28.8) |
| 1. The MHO population has the same risk of developing diabetes as the metabolically healthy normal-weight population. (F) | 122 (22.8) | 252 (47.2) | 160 (30.0) |
| 1. Compared to those with metabolically unhealthy obesity (MUO), individuals with MHO have relatively favorable metabolic characteristics but are still associated with many important chronic diseases, such as cardiovascular diseases, hypertension, type 2 diabetes, chronic kidney disease, and certain types of cancer. (T) | 339 (63.5) | 33 (6.2) | 162 (30.3) |
| 1. Compared to MUO children/adolescents, MHO children/adolescents typically consume more whole grains and polyunsaturated fats and consume less sugary beverages, fried foods, fast food, and processed convenience foods. (T) | 334 (62.5) | 41 (7.7) | 159 (29.8) |
| 1. Maximizing the growth of children and adolescents at a reasonable growth rate is key to controlling MHO. (T) | 369 (69.1) | 21 (3.9) | 144 (27.0) |
| 1. Stratifying obesity in obese children/adolescents based on metabolic status can guide personalized obesity treatment and risk reduction. (T) | 398 (74.5) | 13 (2.4) | 123 (23.0) |

**Table S4.** Attitude dimension

| Item | a. Strongly Agree | b. Agree | c. Neutral | d. Disagree | e. Strongly Disagree |
| --- | --- | --- | --- | --- | --- |
| 1. I believe it’s not normal for children and adolescents in the growth and development stage to be obese, especially in the case of Metabolically Healthy Obesity (MHO). (N) | 31 (5.8) | 73 (13.7) | 110 (20.6) | 260 (48.7) | 60 (11.2) |
| 1. I believe that MHO increases the risk of chronic diseases in adulthood for children and adolescents. (P) | 180 (33.7) | 267 (50.0) | 69 (12.9) | 16 (3.0) | 2 (0.4) |
| 1. I believe it is necessary to control and improve the diet of children and adolescents to manage MHO. (P) | 241 (45.1) | 224 (41.9) | 59 (11.0) | 9 (1.7) | 1 (0.2) |
| 1. I believe it is necessary to control and improve the physical activity of children and adolescents to manage MHO. (P) | 257 (48.1) | 201 (37.6) | 56 (10.5) | 18 (3.4) | 2 (0.4) |
| 1. I’m concerned that managing MHO or MHO itself might affect the normal growth and development of children and adolescents, including their height and weight. (P) | 179 (33.5) | 185 (34.6) | 98 (18.4) | 65 (12.2) | 7 (1.3) |
| 1. I believe MHO will resolve naturally or transform into metabolically healthy, normal-weight individuals as children and adolescents develop. (N) | 24 (4.5) | 44 (8.2) | 217 (40.6) | 223 (41.8) | 26 (4.9) |
| 1. I believe screening for MHO in children and adolescents should be conducted. (P) | 217 (40.6) | 236 (44.2) | 74 (13.9) | 5 (0.9) | 2 (0.4) |
| 1. I believe it is necessary to take MHO children and adolescents to a reputable hospital for medical consultation. (P) | 251 (47.0) | 203 (38.0) | 74 (13.9) | 4 (0.7) | 2 (0.4) |

**Table S5. Practice dimension**

| Item | a. Always | b. Often | c. Sometimes | d. Occasionally | e. Never |
| --- | --- | --- | --- | --- | --- |
| 1. I will control the total food intake of MHO children and adolescents, adjust their dietary structure, and modify their eating behaviors. (P) | 134 (25.1) | 190 (35.6) | 166 (31.1) | 32 (6.0) | 12 (2.2) |
| 1. I will educate MHO children and adolescents to eat at a moderate pace and avoid using electronic devices while eating. (P) | 212 (39.7) | 200 (37.5) | 96 (18.0) | 19 (3.6) | 7 (1.3) |
| 1. I will limit the daily screen time of MHO children and adolescents to within 2 hours. (P) | 220 (41.2) | 189 (35.4) | 91 (17.0) | 26 (4.9) | 8 (1.5) |
| 1. I will encourage MHO children and adolescents to engage in physical activities primarily focused on aerobic and resistance exercises. (P) | 167 (31.3) | 189 (35.4) | 132 (24.7) | 37 (6.9) | 9 (1.7) |
| 1. I will educate MHO children and adolescents to develop healthy sleep hygiene habits, such as maintaining a regular sleep schedule and avoiding stimulating activities before bedtime. (P) | 201 (37.6) | 222 (41.6) | 82 (15.4) | 25 (4.7) | 4 (0.7) |
| 1. If I observe sleep disturbances in MHO children and adolescents, I will take them to a hospital for sleep disorder intervention. (P) | 156 (29.2) | 152 (28.5) | 125 (23.4) | 49 (9.2) | 52 (9.7) |
| 1. I will communicate with MHO children and adolescents to prevent them from developing various negative attitudes due to obesity. (P) | 152 (28.5) | 193 (36.1) | 130 (24.3) | 39 (7.3) | 20 (3.7) |
| 1. I will collaborate with teachers and healthcare professionals to participate in intervention programs for MHO children and adolescents. (P) | 139 (26.0) | 146 (27.3) | 126 (23.6) | 74 (13.9) | 49 (9.2) |
